# Preoperative diagnosis and prediction of microvascular invasion in hepatocellularcarcinoma by ultrasound elastography

**DOI:** 10.1186/s12880-022-00819-0

**Published:** 2022-05-13

**Authors:** Chengchuan Xu, Dong Jiang, Bibo Tan, Cuiqin Shen, Jia Guo

**Affiliations:** 1 Department of Ultrasound, Eastern Hepatobiliary Surgery Hospital Affiliated to Naval Medical University, Shanghai, China; 2 Jiading Branch of Shanghai First People’s Hospital, Shanghai, China

**Keywords:** Hepatocellular carcinoma, Microvascular invasion, Chronic hepatitis B, Elastography

## Abstract

**Background:**

To assess the values of two elastography techniques combined with serological examination and clinical features in preoperative diagnosis of microvascular invasion in HCC patients.

**Methods:**

A total of 74 patients with single Hepatocellular carcinoma (HCC) were included in this study. Shear wave measurement and real-time tissue elastography were used to evaluate the hardness of tumor-adjacent tissues and tumor tissues, as well as the strain rate ratio per lesion before surgery. According to the pathological results, the ultrasound parameters and clinical laboratory indicators related to microvascular invasion were analyzed, and the effectiveness of each parameter in predicting the occurrence of microvascular invasion was compared.

**Results:**

33/74 patients exhibited microvascular invasion. Univariate analysis showed that the hardness of tumor-adjacent tissues (*P* = 0.003), elastic strain rate ratio (*P* = 0.032), maximum tumor diameter (*P* < 0.001), and alpha-fetoprotein (AFP) level (*P* = 0.007) was significantly different in the patients with and without microvascular invasion. The binary logistic regression analysis showed that the maximum tumor diameter (*P* = 0.001) was an independent risk factor for predicting microvascular invasion, while the hardness of tumor-adjacent tissues (*P* = 0.028) was a protective factor. The receiver operating characteristic (ROC) curve showed that the area under the curve (AUC) of the hardness of tumor-adjacent tissues, the maximum diameter of the tumor, and the predictive model Logit(P) in predicting the occurrence of MVI was 0.718, 0.775 and 0.806, respectively.

**Conclusion:**

The hardness of tumor-adjacent tissues, maximum tumor diameter, and the preoperative prediction model predict the occurrence of MVI in HCC patients.

## Introduction

Hepatocellular carcinoma (HCC) accounts for over 80% of primary liver cancer throughout the world and is the fourth leading cause of global cancer-related deaths [1]. Even in acceptable surgery candidates, the long-term survival is unsatisfactory due to the high recurrence rate [2]. Hepatectomy and liver transplantation have been considered as the most effective treatment measurements for HCC. However, the frequent vascular invasion induces intra- and extra-hepatic metastases, and thus the 5-year recurrence rate after surgical resection was > 50% in HCC patients [3, 4].

Reportedly, that microvascular invasion could occur in about 15–57% of HCC patients. MVI is a factor related to the early recurrence of HCC [5], which led to the poor long-term survival of patients after hepatectomy. The occurrence of MVI is associated with tumor metastasis, recurrence, and poor prognoses in HCC patients. As MVI could influence the regimens of local therapy and chemotherapy, the surgical treatments were modified accordingly [6]. Therefore, the preoperative detection of MVI could help in making treatment decisions. However, no accurate preoperative predictive measurements are yet available. Several studies are ongoing on the preoperative prediction of the occurrence of MVI in HCC and the related risk factors, including tumor characteristics, serum biomarkers of the tumor, imaging characteristics, and gene signature. Developing a clinical predictive model has become a research hotspot and a new strategy in predicting MVI based on the combination of various factors [7, 8].

A two-dimensional (2D) ultrasound examination is the first-line a convenient and noninvasive examination imaging method for screening and monitoring of HCC in clinical practice. It could be applied to measure the size of HCC, which is a dominant factor for tumor staging and treatment selection [9–11]. However, the diagnostic value of the 2D ultrasound for focal liver lesions is limited in clinical practice. In recent years, elastography-based ultrasound techniques have gained increasing attention, which could be used for noninvasive assessment of the mechanical properties of tissues. Shear wave measurement (SWM) and real-time tissue elastography (RTE) are two different elastography techniques using based on the excitation methods. SWM utilizes the dynamic stress generated by the ultrasonic transducer to acquire the velocity of shear wave transduction in tissues, which in turn reflected the hardness of the tissues. The ultrasonic transducer of RTE calculates the degree of tissue distortion induced by internal physiological movements to assess the strain ratio (SR), the qualitative measurement of the hardness of the lesion tissues surrounding the normal tissues [12, 13]. The RTE technique was developed before SWM, which could evaluate organs with deep locations without relying on the spherical compression wave on the tissue surface [14]; while the SWM technique could directly provide the quantitative measurements of the hardness of the target tissues. The elastography techniques acquire the qualitative and quantitative data of the changes of liver tissue hardness under pathological status for further diagnosis. In recent years, significant research findings have been obtained on the noninvasive evaluation of liver fibrosis and cirrhosis by elastography techniques. However, only a few studies have investigated the characteristics of focal liver lesions to date [15–17]. Whether the hardness of lesion tissues and normal liver tissues could provide reference information for HCC patients accompanied with MVI is yet unknown.

The present study aimed to analyze the hardness of tumor-adjacent tissues and tumor tissues, as well as the strain rate ratio before surgery using SWM and RTE, respectively, which was then used in combination with serological examination findings and clinical features to investigate the preoperative predictive values on MVI in HCC patients.

## Materials and methods

### Subjects

Data of the 74 patients admitted to our Hospital between January 2020 and July 2021 were analyzed in this retrospective study.

The inclusion criterion was that the patients had single HCC, as assessed by pathological examination following surgical resection.

The exclusion criteria were as follows: (1) HCC treatment history before current hospitalization; (2) the liver tumor diameter > 8 cm or with embolus in intra-hepatic blood vessels; (3) received anti-viral therapy 3 months before the surgery; (4) definite bacterial infection or trauma within 2 weeks before the surgery; (5) history of hepatectomy or other tumors; (6) no definite pathological diagnosis of MVI; (7) laboratory data were incomplete. The study was approved by the Ethics Committee of our hospital, and informed consent was obtained from all patients.

### Clinical data

The clinical data of the patients, including sex, age, liver function stage, and history, were collected by reviewing the clinical records during hospitalization. All the data were collected by clinicians with > 5 years of clinical experience.

### Equipment

**Fig. 1:**
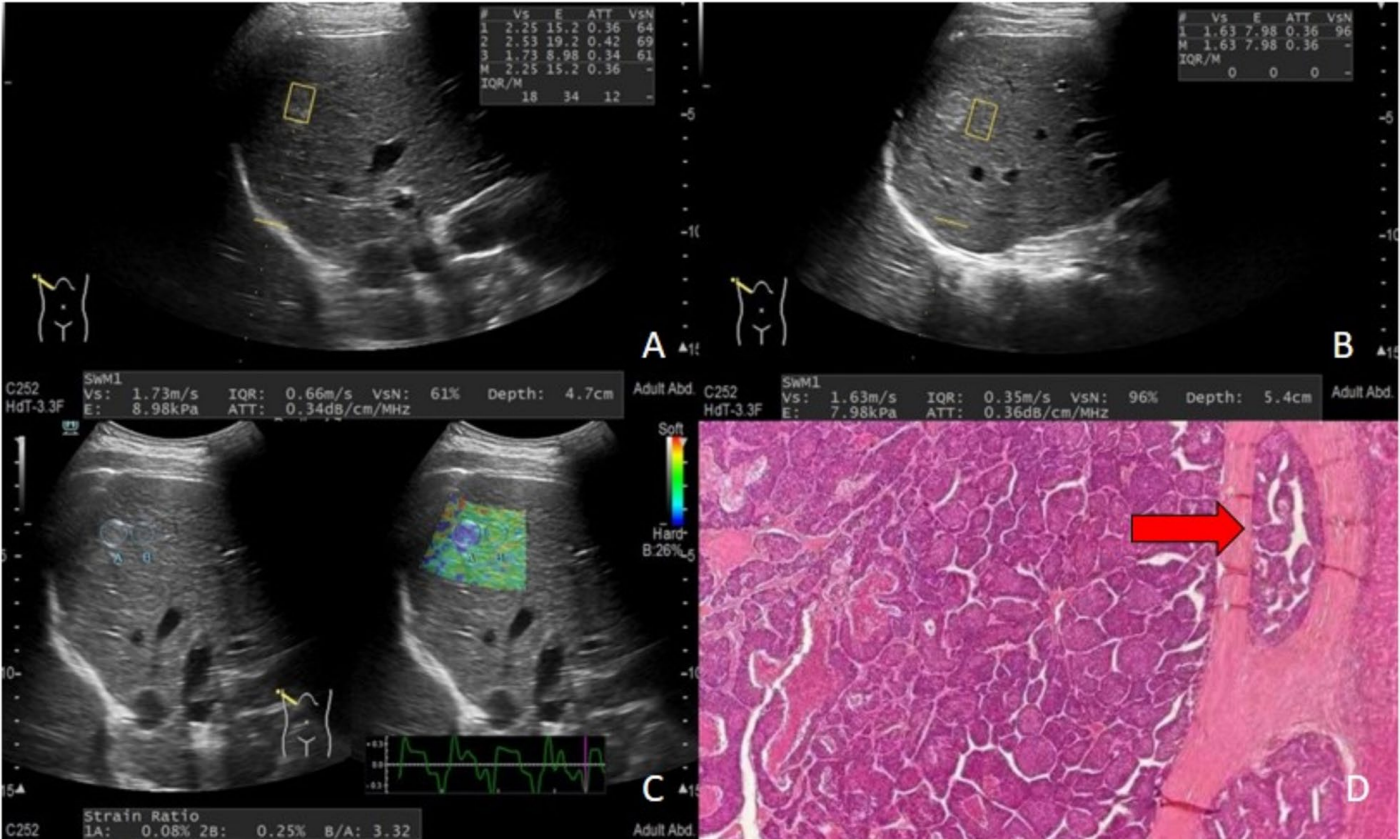
Elastography and pathological examination results of a HCC patient with MVI. **A** Measurementof the hardness of tumor tissues in the SWM mode; **B** Measurement of the hardness of tumor-adjacent tissues in the SWM mode; **C** Measurement of the SR value in the ELASTO mode; **D** Surgical pathology shows crumby cancerous emboli in paratumor small veins (HE × 100)

Hitachi ARIETTA 850 color ultrasound diagnostic instrument and C252 probe were used for ultrasound examination at the frequency of 1–6 MHz. The instrument was equipped with SWM and RTE software. The processes were as follows:

1. Based on the gray-scale ultrasound of the liver, the number of lesions was recorded, and the size of the liver tumors was measured.
2. SWM examination: The patients fasted for > 8 h before the examination. The patients were placed in a supine position with both hands placed near the head to expose the intercostal space. Gray-scale ultrasound examination was performed to observe the maximal cross-section of the lesion. The probe was maintained in parallel to the liver capsule, and the duct structures in the liver were avoided in the right liver lobe (5th, 8th, or 7th segment). The depth of examination was < 5 cm, and the distance to the liver capsule was > 1 cm. The patients were asked to hold their breath in eupnea during the examination. In the SWM mode, the region of interest (ROI) (1 × 1.5 cm) was marked at the margin of the liver tumor, and then the net amount of effective shear wave velocity (VsN) > 60% was set as the reference to assess the hardness of the tumor-adjacent tissues. The same method to measure the hardness of liver tumors. The measurement was performed ten times, and the median was collected for analysis (Fig. 1).
3. RTE examination: The patients fasted for > 8 h before the examination. For the examination, the patients were placed in the supine position, with both hands near the head to expose the intercostal space. Gray-scale ultrasound examination was performed to observe the maximal cross-section of the lesion. The probe was maintained parallel to the liver capsule, and the duct structures in the liver were avoided in the right liver lobe (5th, 8th, or 7th segment). The depth of examination was < 5 cm, and the distance to the liver capsule was > 1 cm. The patients were asked to hold their breath in eupnea during the examination. In the ELASTO mode, the liver tumor was placed in the ROI frame of elastography, and several frames of elastography pictures were acquired when three continuous normal liver histograms appeared in the strain diagram. The area of the valley in the histogram was selected as the reference for the selection of the sampling frame of the static image, and the software equipped by the instrument was used to calculate the strain rate. The total liver tumor region was selected as ROI A, and the normal liver tissues at the tumor margin were selected as ROI B: both at the same depth. The ratio of the strain rate of ROI B to ROI A was calculated as the SR, which was measured five times, and the median was calculated for analysis. All the ultrasound examinations were performed by the same sonographer who had received professional training previously. (Fig. 1).
4. Laboratorial examination: Fasting venous blood was obtained from all the patients 3 days before the surgery. The cytological examination for blood was performed by the BC-5800 automatic hematology analyzer, and liver functions were measured by the AU800 automatic biochemical analyzer. Subsequently, total bilirubin (TBil), white blood cells (WBCs), red blood cell distribution width (RDW), alpha fetoprotein (AFP), protein induced by vitaminK absence or antagonist- II (PIVKA-II), platelets (PLTs), aspartate transaminase(AST), alanine aminotransferase (ALT), prothrombin time(PT), albumin(ALB), γ-glutamyltransferase(GGT), alkaline phosphatase (ALP), and glucose (Glu) were recorded.
5. Pathological examination: For the specimens collected by hepatectomy, tumor sections with complete tissues and less bleeding and necrosis were selected. At least four specimens were acquired at 12, 3, 6, and 9 o’clock at the junction of the tumor and adjacent liver tissues.MVI was defined as the nesting of cancer cells in the vascular lumen, which mainly occurred in the branches of the portal vein. The risks of MVI were classified into three grades, including MVI-negative (no MVI), low-risk MVI-positive (number of MVI < 5 and MVI in adjacent peritumoral liver tissues (≤ 1 cm from the tumor capsule)), and high-risk MVI-positive (MVI in distant peritumoral liver tissues (> 1 cm from the tumor capsule), or number of MVI > 5) [18]. The tumor number, Edmondson–Steiner grade, MVI state, and pathological features of parenchymal cirrhosis were independently evaluated by two experienced pathologists.

### Statistical analysis

SPSS 22.0 (SPSS, Inc., Chicago, IL, USA) was used for statistical analysis. Quantitative data in normal distribution were described as mean ± standard deviation and compared using an independent *t*-test if the variance of data was homogeneous. Quantitative data in non-normal distribution were described with medians and interquartile ranges (IQR) and compared using Mann–Whitney U test. Qualitative data were described by frequencies and percentages and compared using the chi-square test. The variables with statistical significances in univariate analysis were included in the multivariate binary logistic regression analysis to explore the risk factors of MVI states. The receiver operator characteristic curve (ROC) was plotted, and the area under the curve (AUC) was estimated and compared with the Delong test. P < 0.05 indicated statistical significance.

## Results

### General and clinical characteristics of patients

The cohort consisted of 74 patients (60 males and 14 females) with single HCC, proven by surgical pathology. The median age of the patients was 57 (49–62) years. 61/74 (82.4%) patients had hepatitis B virus (HBV) infection, 35/74 (47.3%) patients had background liver cirrhosis, 67/74 (90.5%) patients exhibited Child–Pugh grade A liver function, and 7/74 (9.5%) patients had Child–Pugh grade B liver function. 33/74 (44.6%) patients presented MVI, while the remaining 41/74 (55.4%) patients did not have MVI, and the Edmondson–Steiner grade was I, II, and III, in 2, 2 and 70 patients, respectively. The age, sex, Edmondson–Steiner grade, HBV infection, background liver cirrhosis, Child–Pugh stage, tumor hardness, WBC, PLT, RDW, PT, ALB, ALT, AST, GGT, TB, ALP, and Glu did not differ significantly between the two groups. In contrast, maximum tumor diameter (*P* = 0.001), AFP level (*P* = 0.006), PIVKA-II level (*P* = 0.026), tumor-adjacent tissue hardness (*P* = 0.001), and SR (*P* = 0.023) were significantly different between the patients with and without MVI (Tables 1 and 2).

**Table 1:** General clinical data of the patients

Characteristics	Total	With MVI (n = 33)	Without MVI (n = 41)	*P*-value
Age (y)	57 (49–62)	53 (49–57)	59 (55–62)	0.060
Sex				0.297
Male	60	25 (41.6)	35 (58.4)	
Female	14	8 (57.2)	6 (42.8)	
Maximum tumor diameter (mm)	54.72 (48.89–61.73)	70.15 (60.41–80.10)	42.29 (36.33–48.85)	0.001
Edmondson–Steiner grade				0.835
Grade I	2 (2.7)	1 (3.0)	1 (2.4)	
Grade II	2 (2.7)	1 (3.0)	1 (2.4)	
Grade III	70 (94.5)	31 (94.0)	39 (95.1)	
Etiology				0.626
HBV +	61 (82.4)	28 (84.8)	33 (80.5)	
HBV −	13 (17.6)	5 (15.2)	8 (19.5)	
Background liver cirrhosis				0.266
Yes	35 (47.3)	18 (54.5)	17 (41.5)	
No	39 (52.7)	15 (45.5)	24 (58.5)	
Child–Pugh stage				0.460
A	67(90.5)	30(90.9)	37(90.2)	
B	7(9.5)	3(9.1)	4(9.8)

**Table 2:** Ultrasound and laboratory examination results of patients

	Total	With MVI	Without MVI	*P*-value
Tumor-adjacent tissue hardnss	13.46 ± 5.72	11.16 ± 5.63	15.32 ± 5.14	0.001
Tumor hardnss	11.66 ± 8.16	12.95 ± 7.33	10.62 ± 8.72	0.225
SR	1.85 ± 1.27	1.48 ± 0.89	2.15 ± 1.45	0.023
AFP	16.92 (1–1210)	363.10 (2–1210.0)	5.93 (1–1210)	0.006
PIVKA-II	252 (0–64, 199)	958 (0–64, 199)	99 (0–16, 602)	0.026
WBC	5.17 ± 1.95	5.04 ± 1.93	5.28 ± 1.99	0.600
PLT	167.62 ± 66.93	176.15 ± 62.10	160.76 ± 70.58	0.329
RDW	12.55 (11.3–19.5)	12.4 (11.5–19.5)	12.6 (11.3–17.7)	0.304
PT	11.5 (10.0–15.3)	11.5 (10.2–13.1)	11.5 (10.0–15.3)	0.446
ALB	41.69 ± 3.61	41.0 ± 3.59	42.19 ± 3.58	0.342
ALT	26 (11–91)	26 (12–74)	27 (11–91)	0.433
AST	26 (11–112)	27 (11–101)	26 (15–112)	0.325
GGT	39.5 (11–210)	41 (11–210)	39 (11–161)	0.253
TB	12.9 (5.1–40.5)	14.3 (6.5–40.5)	12.3 (5.1–30.6)	0.064
ALP	81 (38–347)	84 (38–347)	80 (38–187)	0.691
Glu	5.16 (3.33–16.26)	5.13 (3.57–14.01)	5.19 (3.33–16.26)	0.539

### Value of parameters in preoperative prediction of MVI

The results of univariate regression analysis are shown in Tables 3. The maximum tumor diameter (odds ratio (OR) = 1.044, 95% confidence interval (CI): 1.021–1.069, *P* < 0.001), AFP level (OR = 1.001, 95% CI:1.000–1.002, *P* = 0.007), tumor-adjacent tissue hardness (OR = 0.864, 95% CI: 0.784–0.952, *P* = 0.003), and SR (OR = 0.610, 95% CI: 0.389–0.957, *P* = 0.032) were significant preoperative risk factors associated with MVI in the univariate analysis. Then, these four parameters were included in the multivariate logistic regression analysis, which showed that maximum tumor diameter (*P* = 0.001) was a risk factor predicting MVI, and tumor-adjacent tissue hardness (*P* = 0.028) was a protective factor of MVI (Table 4).

**Table 3:** Univariate regression results of parameters for preoperative prediction of MVI state

Parameter	OR (95% CI)	*P*-value
Age (years)	0.964 (0.927–1.002)	0.064
Sex	1.867 (0.576–6.053)	0.298
HBV infection	1.358 (0.399–4.624)	0.625
Background liver cirrhosis	1.032 (0.802–1.328)	0.807
Maximum tumor diameter	1.044 (1.021–1.069)	< 0.001
Edmondson–Steiner grade	0.872 (0.244–3.111)	0.833
Child–Pugh stage	2.920 (0.320–28.082)	0.325
Tumor-adjacent tissue hardness	0.864 (0.784–0.952)	0.003
Tumor hardness	1.036 (0.978–1.098)	0.226
SR	0.610 (0.389–0.957)	0.032
AFP	1.001 (1.000–1.002)	0.007
PIVKA-II	1.000 (1.000–1.000)	0.095
WBC	0.936 (0.734–1.194)	0.596
PLT	1.004 (0.997–1.011)	0.325
RDW	1.011 (0.732–1.396)	0.947
PT	0.753 (0.448–1.267)	0.285
ALB	0.939 (0.826–1.068)	0.339
ALT	0.989 (0.960–1.019)	0.482
AST	1.011 (0.985–1.038)	0.418
GGT	1.008 (0.998–1.019)	0.131
TB	1.073 (0.987–1.167)	0.100
ALP	1.005 (0.993–1.017)	0.410
Glu	0.874 (0.680–1.124)	0.295

**Table 4:** Multivariate regression results of parameters for preoperative prediction of MVI state

Parameter	B	OR (95% CI)	*P*-value
Tumor-adjacent tissue hardness	− 0.123	0.884 (0.792–0.987)	0.028
Maximum tumor diameter	0.040	1.041 (1.016–1.066)	0.001
Constant	− 0.810	0.45	

### Construction of MVI predictive model and comparison of diagnostic power of parameters for MVI

According to the findings of multivariate regression analysis, the regression equation “Logit(P-0.810–0.123 × tumor-adjacent tissue hardness + 0.04 × maximum tumor diameter” was acquired. The Logit(P) 's accuracy is 75.7%.

**Fig. 2:**
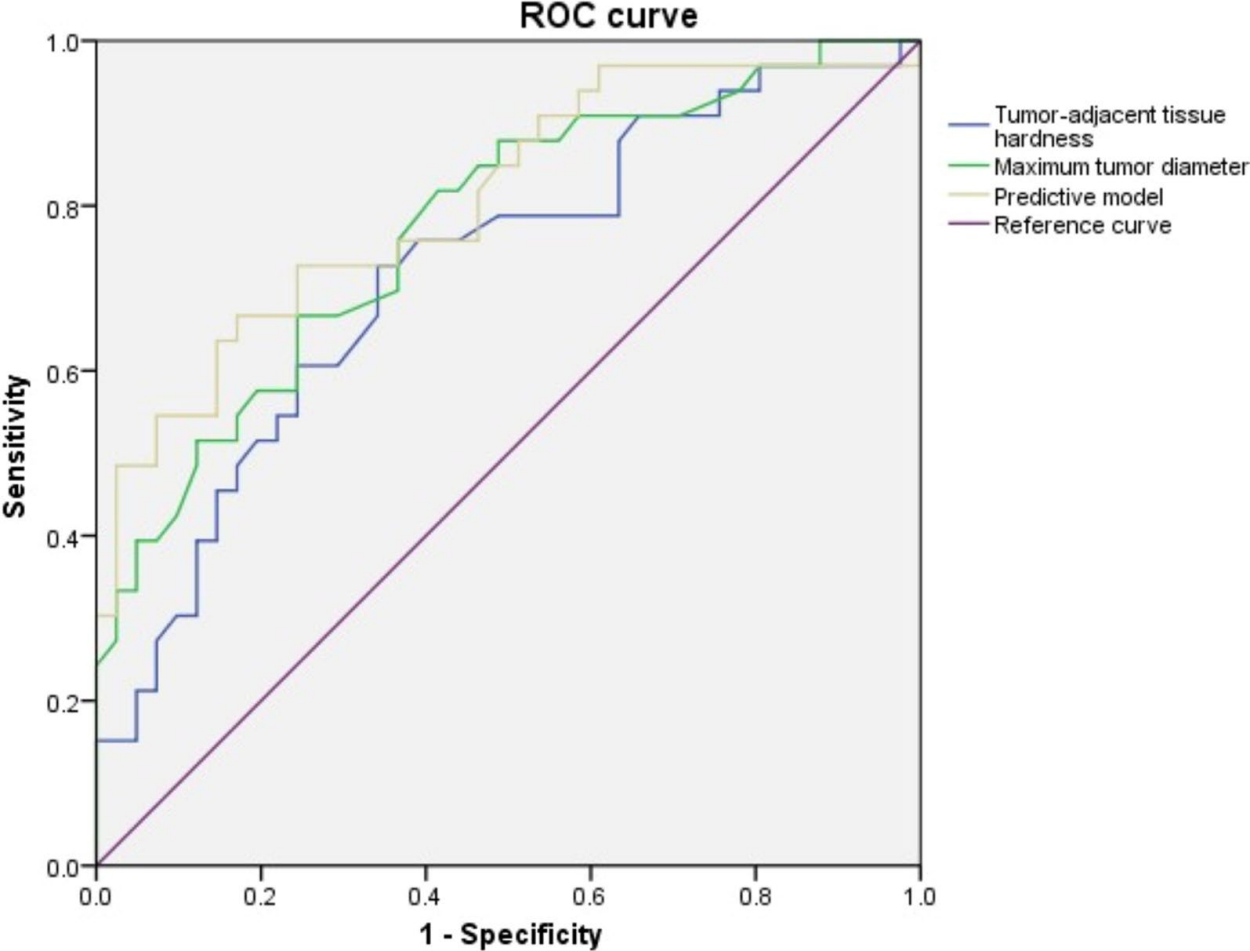
ROC curves of different parameters

The AUC of tumor-adjacent tissue hardness was 0.718 (95% CI: 0.600–0.836, *P* = 0.001), the cutoff value was 14.150, sensitivity was 0.727, and specificity was 0.659. The AUC of maximum tumor diameter was 0.775 (95% CI: 0.668–0.881, *P* < 0.001), the cutoff value was 43.50, sensitivity was 0.818, and specificity was 0.585 (Table 5, Fig. 2).

**Table 5:** comparison of MVI predictive model and parameters diagnostic power for MVI state

Parameter	AUC	95% CI	Cutoff value	Sensitivity	Specificity
Tumor-adjacent tissue hardness	0.718	0.600–0.836	14.150	0.727	0.659
Maximum tumor diameter	0.775	0.668–0.881	43.50	0.818	0.585
Logit(P)	0.806	0.705–0.907	0.68	0.485	0.976

## Discussion

MVI is common in patients with HCC, which could reflect the high invasiveness of HCC and the capacity of metastasis in the early stage. The incidence of MVI is > 20% even in small HCC (< 3 cm) [19, 20]. MVI is a major risk factor for low postoperative survival rate and contributes to postoperative tumor recurrence. The *Guidelines for the Diagnosis and Treatment of Hepatocellular Carcinoma (2019 Edition)* have stressed that MVI is crucial to evaluate the probability of HCC recurrence and select the treatment strategy, and thus should be applied routinely in pathological examinations [18]. In this study, the incidence of MVI in the 74 patients was almost 44.6%, and the incidence of small hepatocellular carcinoma (SHCC) was 4.05%. The existence of MVI could influence the selection of surgical methods and the outcomes of surgery. Cucchetti et al. [21]showed that for early HCC patients with MVI or poorly differentiated early HCC patients, non-anatomical resection increases the early recurrence. To date, several clinical predictive models that included clinical characteristics, laboratory parameters, and imaging characteristics have been constructed [22–24], which accurately predict MVI. However, only a few studies have applied elastography techniques for the preoperative prediction of MVI in HCC patients.

Elastography could be used for the noninvasive evaluation of the mechanical characteristics of tissues. RTE is a technique that evaluates the tissue distortion caused by the compression on tissues via external forces and generates the strain elasticity diagram to illustrate the relative hardness of the tissues. The SR is calculated by dividing the strain rate of adjacent reference tissues by the strain rate of the examined tissues. A high SR indicates the high hardness of the examined tissues. The calculation of SR is not restricted by the external forces utilized in the examination, and thus SR has been widely used in clinical practice [25]. However, RTE techniques have several major restrictions, including high dependence on the skill of the operators, low repeatability, and obtaining only qualitative or semi-quantitative information [26, 27]. The shear wave measurement (SWM) technique utilizes acoustic radiation force impulse to transmit controllable longitudinal forces, which deform the tissues and generate transverse shear waves, and the transducer detects the shear wave velocity (SWV) to measure the tissue stiffness. Compared to RTE, the advantages of SWM are less operator dependence and quantitative SWV measurements [28]. The univariate analysis in this study showed that tumor-adjacent tissue hardness and SR were significantly different between the patients with and without MVI, and tumor-adjacent tissue hardness was a protective factor for MVI. The appearance of tumor cells in microvessels of tumor-adjacent tissues reduces the number of blood cells in the microvessels, modifies the tissue hardness, and consequently changes the tumor-adjacent tissue to tumor tissue SR. Previous studies suggested that liver fibrosis is an independent risk factor of SWV measurements [29], and the hardness of tumor-adjacent tissues was significantly higher in patients with liver fibrosis than in those without liver fibrosis. The incidence of MVI was 44.6%, which was not ubiquitous in the patients, and thus it could be speculated that the hardness of tumor-adjacent tissues might be influenced by liver fibrosis.

The univariate analysis in this study showed that serum AFP level was significantly different between patients with and without MVI, while the findings of multivariate analysis suggested that AFP was not an independent risk factor of MVI. The current findings showed that PIVKA-II and Edmondson–Steiner grades did not differ significantly between the two groups, which was not in agreement with previous studies [30–32]. We speculated that the inconsistency could be caused by the small sample size.

The findings of this study showed that maximum tumor diameter was an independent risk factor of MVI, and higher maximum tumor diameter indicated elevated invasiveness to adjacent tissues, which was in agreement with the findings of previous studies [8].

Nevertheless, the present study has several limitations. Firstly, the sample size of the study was small. Secondly, all the data were obtained from one institute; thus, additional data from other institutes are needed to verify the reliability of the model. Thirdly, selection bias could exist in this study due to the single-center retrospective study design. Finally, the hardness of tumor-adjacent tissues has not been investigated before, which might involve various influencing factors and requires sophisticated analyses in future studies.

## Conclusion

Herein, SWM included two methods, these are pSWE and 2D SWE, the results of this study are obtained with Pswe. We developed a preoperative prediction model for MVI in patients with HCC. With the inclusion of two tumor features (tumor-adjacent tissue hardness and maximum tumor diameter), our prediction model could differentiate between HCC patients with and without MVI with an accuracy of 75.7% However, these findings need to be verified further.

## Data Availability

The datasets used and/or analysed during the current study are available from the corresponding author on reasonable request.

## Abbreviations

ROC: Receiver operating characteristic
AUC: Area under the curve
HCC: Hepatocellular carcinoma
RTE: Real-time tissue elastography
SR: Strain ratio
ROI: Region of interest
TBil: Total bilirubin
WBCs: White blood cells
RDW: Red blood cell distribution width
AFP: Alpha fetoprotein
PIVKA-II: Protein induced by vitaminK absence or antagonist- II
PLTs: Platelets
AST: Aspartate transaminase
ALT: Alanine aminotransferase
PT: Prothrombin time
ALB: Albumin
GGT: γ-Glutamyltransferase
ALP: Alkaline phosphatase
Glu: Glucose
IQR: Interquartile ranges
SHCC: Small hepatocellular carcinoma
